# Concurrent Eosinophilia Increases the Prevalence of Nail Abnormalities and Severity of Hair Loss in Patients With Alopecia Areata

**DOI:** 10.1155/2024/5596647

**Published:** 2024-09-07

**Authors:** Giovanni Damiani, Laura Cristina Gironi, Rosalynn R. Z. Conic, Massimo del Fabbro, Paola Savoia, Marco Fiore, Wilma F. Bergfeld

**Affiliations:** ^1^ Italian Center of Precision Medicine and Chronic Inflammation University of Milan, Milan, Italy; ^2^ Department of Biomedical Surgical and Dental Sciences University of Milan, Milan, Italy; ^3^ AOU Maggiore della Carità, Novara, Italy; ^4^ Department of Dermatology Cleveland Clinic, Cleveland, Ohio, USA; ^5^ Fondazione IRCCS Ca' Granda Ospedale Maggiore Policlinico, Milan, Italy; ^6^ Department of Health Sciences University of Eastern Piedmont, Novara, Italy; ^7^ Department of Women Child and General and Specialized Surgery University of Campania “Luigi Vanvitelli”, Naples, Italy

**Keywords:** alopecia, Alopecia areata, atopic dermatitis, atopy, eosinophilia, eosinophilic disease

## Abstract

**Background:** The potential link between alopecia areata (AA) and eosinophilia is unclear, as well as its clinical manifestations in these patients' subsets.

**Methods:** This is a monocentric retrospective observational study in which clinical and laboratory data were summarized and evaluated the AA subset with concurrent eosinophilia.

**Results:** In a sample of 205 AA patients, 38 (18.5%) were classified as AA with eosinophilia. Interestingly, this subset of patients had a statistically higher prevalence of atopia and nail abnormalities (*p* < 0.05) than AA without eosinophilia. AA patients with eosinophilia had a 3.70 higher odds of more severe hair loss versus age- and gender-matched AA without eosinophilia.

**Conclusions:** AA patients with eosinophilia had distinctive clinical and laboratory characteristics, so future studies may potentially explore the use of IL-5 inhibitors.

## 1. Introduction

Alopecia areata (AA) is a nonscarring hair loss disorder that ranges in presentation from (i) single delimited patches of hair loss (patchy AA); (ii) multiple coalescing patches on the scalp (diffuse patchy AA) and/or body (extensive patchy AA); and (iii) total loss of scalp (totalis AA, AT) or body (universalis AA, AU) hair [1, 2]. Though the pathogenesis of AA remains poorly understood, currently AA is thought to involve an autoimmune attack of anagen hair follicles that have lost immune privilege [1]. However, it remains unclear what factors act on this common pathogenetic mechanism and leads to a widely variable phenotypic expression of hair loss.

A number of comorbid conditions have been reported in association with AA [3, 4]. Atopy has been observed in more than 40% of AA patients, which is twice the prevalence in the general population [2, 5]. Atopic conditions, in particular atopic dermatitis (AD), have been previously reported to be associated with more severe phenotypes of AA, such as AT and/or AU [6]. In contrast, eosinophilia has been found most commonly and robustly in diffuse AA, a recently identified subtype with broad scalp hair loss that typically regrows fully within 1 year [7]. Nevertheless, previous studies have identified patients with eosinophilia in conjunction with AT or AU.

Given that eosinophilia is often considered a characteristic of atopic processes, it is interesting that eosinophilia and atopy appear differentially distributed among AA subtypes. It is also unclear whether instances of eosinophilia in AA are secondary to underlying atopy or appear as an independent phenomenon [6–9]. Noteworthy, patients with AD and concurrent AA may benefit also from AD-dedicated target therapies that preferentially inhibit the IL-4/IL-13 axis [10–12].

Thus, the aim of this study was to determine the prevalence of eosinophilia and atopy in AA, as well as their coexistence, and to evaluate their relationship with disease severity. Based on our clinical experience, we hypothesized that serum, systemic, or lesional eosinophilia would be associated with severe AA.

## 2. Materials and Methods

A retrospective review of medical records of 205 AA patients seen at Cleveland Clinic from 2004 to 2015 and mapped in the hospital database was conducted. This study was approved by the Cleveland Clinic Foundation Institutional Review Board (No. 15-727). All patients were evaluated clinically and dermoscopically (Delta 20, HEINE Optotechnik GmbH & Co. KG, Gilching, Germany) [10, 11] at the Cleveland Clinic Main Campus Department of Dermatology. Demographic factors including race, age, and gender were recorded. The diagnosis of AA was based on clinical features as assessed by hair specialist dermatologists. Patients were categorized into alopecia subtypes based on hair specialist dermatologist classification of the percentage and location of hair loss: focal (i.e., including only eyebrows, beard, or other sites of the face); patchy; ophiasis; diffuse patchy; alopecia totalis (AT); alopecia universalis (AU).

The presence of atopic disease at the time of patients' hair loss evaluation period was recorded. AA patients were classified as atopic (AtAA) if (a) they met Hanifin Criteria [12] or The U.K. Working Party's Diagnostic Criteria for Atopic Dermatitis [13], or if they have a (b) current or past diagnosis of allergic asthma, (c) allergic rhinitis, (d) hyper IgE syndrome, and/or (e) allergic urticaria. The presence of eosinophilia at the time of the patients' hair loss evaluation period was also recorded. Eosinophilia is defined as greater than 500 eosinophils/mm^3^. The degree of eosinophilia can be categorized as mild (500–1500 cells/mm^3^), moderate (1500 to 5000 cells/mm^3^), or severe (> 5000 cells/mm^3^) [14].

Eosinophilic AA patients (EoAA) were defined as those with either two or more elevated serum eosinophil counts or a current or past diagnosis (< 5 years) of systemic eosinophilic disease (i.e., Ofuji disease).

Conversely, AA subjects who were not classifiable neither as AtAA nor as EoAA were defined as control AA patients (CnAA).

Statistical relationships were tested using the Pearson *χ*^2^ test, 2-sample *t*-tests, Kruskal–Wallis test, one-way ANOVA, or proportional odds regression as appropriate. All analysis was performed using JMP statistical software, and all tests were conducted with statistical significance set as *p* < 0.05.

## 3. Results

Table 1 summarizes all the demographic and clinical data of our AA patients, no pathognomonic dermatoscopic patterns were found. All AA patients were biopsied.

Among the 205 AA patients, 38 (18.5%) were classified as eosinophilic (EoAA). Interestingly, 32 of 38 (84.2%) of EoAA patients were qualified based on persistent serum eosinophilia. The remaining 6 patients were identified by eosinophilic infiltrates histologically confirmed on biopsy. Specifically, 2 patients had inflammatory bowel disease, 2 patients had eosinophilic infiltrates on scalp biopsy, 1 patient had pulmonary eosinophilic granuloma, and 1 patient had eosinophilic esophagitis.

Remarkably, 31 of 38 (81.6%) EoAA patients had concomitant atopic conditions. Of the 7 remaining EoAA patients with no atopic disease, 6 were diagnosed with persistent serum eosinophilia, and 1 had a scalp biopsy positive for eosinophilic infiltrate.

Then 92 of 205 (44.9%) AA patients were classified as atopic without eosinophilia (AtAA). The prevalence of specific atopic conditions was compared across the atopic (AtAA) and eosinophilic (EoAA) groups. There was no significant difference in the prevalence of all other atopic conditions across the two groups. The most common atopic conditions across both groups were allergic rhinitis, AD, and asthma, respectively.

In all the groups analyzed, the female gender and the Caucasian ethnicity were the most represented demographic features. AA was diagnosed more frequently in young adults (between the third and fourth decade of life), with no significant differences between groups.

Personal and family history for both autoimmune diseases and AA family members were recorded in each group analyzed, without statistically significant differences. In particular, 37 patients had concurrent Hashimoto thyroiditis, 14 psoriasis, 9 Inflammatory bowel disease, 8 spondylitis, 5 Behcet disease, 3 rheumatoid arthritis, and 1 hidradenitis suppurativa.

Nail abnormalities were significantly more frequent in the EoAA group (39.5% of 38 patients), compared to CnAA and AtAA (19% of 75 patients and 9.8% of 92 patients, respectively). The main nail abnormalities detected were chronic paronychia (81.0%) and pitting (37.0%).

EoAA patients had a 3.70 higher odds of more severe hair loss versus age- and gender-matched CnAA (Table 2). After adjustment for comorbid autoimmune disease and family history of AA or autoimmune disease, the odds ratio for severe disease decreased to 3.45 but remained significantly greater than the odds of severe disease for AA controls (*p* < 0.01). A statistically significant increase in the odds of severe disease was also observed for AtAA patients against CnAA, with unadjusted and adjusted odds ratios of 2.33 and 2.31, respectively (*p* < 0.01). Conversely, there was no significant difference in the odds of severe disease between EoAA and AtAA patients (*p* = 0.20 unadjusted, *p* = 0.28 adjusted). In the adjusted proportional odds model, patients with concomitant autoimmune disorders presented with significantly greater severity of hair loss (OR: 2.33; *p* = 0.003).

## 4. Discussion

We report an 18.5% prevalence of eosinophilia in patients suffering from AA that was found to be significantly associated with more severe disease, compared to control patients. AA atopic patients without eosinophilia (AtAA), which represent 44.9% of the considered population, also showed a statistically significant higher frequency of severe disease compared to controls AA subjects.

Although there was a trend toward greater odds of severe disease for EoAA versus AtAA patients, it did not reach the level of statistical significance. Moreover, 81.6% of EoAA patients also had concomitant atopic conditions. As a result, we cannot distinguish whether the association between eosinophilia and severe disease was a function of underlying atopy or an effect of eosinophilia specifically. The relationship between atopy, eosinophilia, and IgE in AA patients remains not yet fully clarified, and this aspect justifies the therapeutic challenge that these patients represent for clinicians.

It has commonly been reasoned, based on similar previous results, that atopy may amplify AA by sharing a Th2 cytokine profile that triggers an “allergic” type reaction involving IgE, eosinophils, and mast cells [15]. In fact, growing evidence suggests that atopy, particularly AD, involves both Th2 and Th1 cytokines, predominately acute and chronic phases, respectively [16]. Several recent researches have characterized the cytokine profile of AA, showing an early regulation of Th2 cytokines in localized AA and a Th1 response in chronic, extensive AA [5, 16, 17]. Interestingly, patients with AA and atopy have also been found to have elevated levels of Th1 cytokines (including IL-1*α*, TNF-*α*, IFN-*γ*, and IL-12), which further emphasizes that both Th1 and Th2 cytokines are relevant. Thus, there are multiple complementary or additive mechanisms when the two conditions coexist that potentially could contribute to greater AA severity in this population.

Moreover, both Th1 and Th2 cytokines associated with atopy and AA can activate eosinophils. Eosinophil activity is well known to be induced by Th2 cytokines, most notably IL-5, and to a lesser degree IL-4 and IL-13 [18]. Eosinophils can also be stimulated by Th1 cytokines such as eotaxin and RANTES. Eotaxin is an eosinophil chemoattractant, and RANTES is atypical in serving as both a chemoattractant for Th1 cells as well as a participant in the Th2 response [19, 20]. Kuwano et al. demonstrated in AA patients an elevation in RANTES levels relative to healthy controls with the greatest magnitude of elevation in acute exacerbations, intermediate levels in stable disease, and the lowest levels with disease regression, suggesting a correlation with AA disease activity. This same study also showed that peripheral eosinophils were more robustly elevated in extensive patchy AA, AT, and AU patients relative to monopatchy AA, though RANTES expression did not vary significantly with increasing severity [9].

Eosinophil-mediated amplification of Th2 cytokines may cause intensification of damage in acute lesions and persistence of chronic disease, through downregulation of filaggrin and keratin gene expression that disrupts hair follicle structure. This mechanism is more prominent in severe disease, as a previous study showed patients with extensive AA had more widespread keratin loss in both lesional and nonlesional areas compared to patchy AA and healthy controls [5, 21].

From the histological point of view, peribulbar lymphocytic infiltrates are often considered the diagnostic histopathologic feature associated with AA; however, it has been noted to be subtle or absent in chronic AA [22]. The presence of peribulbar eosinophils or eosinophils within fibrous tracts has frequently been documented as a finding of interest for diagnostic and subtyping purposes in a subset of 18%–54% of AA patients. Among these cases, some studies have reported, in a variable percentage (between 48% and 20% approximately), a histological subgroup in which peribulbar lymphocytic infiltrate was absent [22–26]. In addition, some authors have noted that eosinophilic infiltrate may be more frequent in the acute stages of AA; whilst in chronic phases, it appears less detectable, similarly to peribulbar lymphocytic infiltrate. It thus remains unclear whether eosinophils appear secondary to the lymphocytic infiltrate or as a result of upstream and/or independent mechanisms.

Additionally, Zhang et al. demonstrated an association between peripheral eosinophilia and an increased number of mast cells in the upper dermis perivascular and deep perifollicular areas in acute AA lesions [27]. This is not unexpected as eosinophil products (e.g., eosinophil-derived granule proteins, eosinophil cationic protein, eosinophil peroxidase, major basic protein) can degranulate mast cells either directly or indirectly by activating IgE for mast cell crosslinking [27]. Signals from mast cells may facilitate crosstalk between CD8+ T-lymphocytes to enhance the regression of hair follicle cycling [27]. Of relevance in chronic disease, peripheral eosinophilia was associated with decreased CD4+ T cell infiltration in the upper perifollicular area [28]. This limits the availability of costimulatory factors to perpetuate autoimmune attacks of the hair follicle, which could contribute to the quiescence or stability of severe AA lesions in the long term. Eosinophils may also stimulate increased IgE production by mast cells and increased B cell activation and class switching to contribute a more chronic humoral autoimmune attack in addition to the primary cell-mediated autoimmune processes [29]. Previous studies have demonstrated a significant increase in mean total IgE levels in AA patients compared to controls and, among AA patients, in those suffering from chronic disease and AU [7, 21, 28–30]. There are several mechanisms by which eosinophils and IgE interact. Eosinophils and IgE can both be independently activated by Th2 cytokine release. IgE crosslinking of mast cell receptors also leads to the release of IL-5, a strong promoter of eosinophil activation. It is well known that eosinophils can express three high-affinity IgE receptors (Fc*ε*RI); moreover, in several human diseases featured by eosinophilia and high IgE levels, mRNA for the Fc*ε*RI chains has been detected in circulating eosinophils [31].

In summary, a review of the literature suggests that eosinophils in AA may potentially be stimulated by one or a combination of Th2 cytokines, Th1 cytokines, and/or IgE.

To date, eosinophilia has been observed primarily in diffuse AA or in more severe phenotypes, such as AT/AU [5, 22]. This may be due to limited investigation of eosinophilia in chronic severe AA. Alternatively, eosinophil-associated mechanisms may peak in the early stages of AA, with the relative severity of subsequent disease determined by the duration and intensity of this response [5, 16]. In either case, the prominence of eosinophils in severe disease in only a subset of AA patients serves as a reminder that the AA population is heterogenous [5, 9, 16]. Severe AA may represent the final common pathway of a stronger, more diverse inflammatory response that can be triggered by a broad range of initiating factors. Eosinophils may be just mere byproducts of the immune response in AA. However, once activated, eosinophils may also contribute to disease pathogenesis of their own accord. Eosinophils release Th2 cytokines (IL-4, IL-5, IL-9, IL-13, and IL-25), Th1 cytokines (IL-12 and IFN-*γ*), proinflammatory cytokines (TNF-*α*, IL-1*β,* IL-6, and IL-8), and inhibitory cytokines (IL-10 and TGF-*β*) [16, 17, 21, 28]. This permits eosinophils to augment the activity of a wide variety of immune mediators.

The strengths of our study include a large population in this rare condition. The history of atopy was confirmed by a combination of physician evaluation, biopsy analysis, and/or laboratory values rather than relying on patient self-reporting. Limitations of this study were performed in a single center secondary to its retrospective nature and potential for recall bias and reporting bias. Moreover, as previously stated, study inclusion criteria required all patients to have a minimum of two complete blood counts with differential tests in order to rule out the presence of persistent eosinophilia, which introduces some level of selection bias. Furthermore, the study did not evaluate mast cells, cytokinetic pattern, and the potential correlation between these parameters and a severity index.

The majority of EoAA patients also had concomitant atopy, which leaves careful questioning about AD, asthma, and allergic rhinitis, in the review of systems as the most cost-efficient method for dermatologists to identify this risk factor. For EoAA patients without comorbid atopy, a history of eosinophilic disease can sometimes be gleaned from pathology reports and may aid risk assessment. Further research is required to define parameters for the use of complete blood count with differential as a screening tool in AA, as our sample of seven nontopic, nonbiopsy-diagnosed eosinophilic patients is insufficient to draw conclusions at this time. We hypothesize that periods of elevated serum eosinophilia may correspond to disease flares or periods of acute worsening.

In conclusion, both persistent serum eosinophilia and atopy are associated with more severe phenotypes of AA, but future multiomics studies should establish if EoAA may represent a distinctive clinical endotype meritful of targeted therapeutic approach (i.e., IL-5 antagonists). Further research is required to determine if eosinophils are active contributors or a byproduct of etiopathogenic mechanisms of AA and to what extent they function independently from atopic processes in AA by understanding the pathomechanism-linking AA and eosinophilia that suggest the more appropriate target therapy for these patients [32–34] and increasing, at the same time, the overall compliance [35–38].

## Figures and Tables

**Table 1:** Demographic and clinical data of our AA patients, stratified by groups (controls with AA, eosinophilic AA, and AA with atopia).

**Factor**	**Control with AA (** **n** ** = 75)**	**Eosinophilic AA (** **n** ** = 38)**	**AA and concurrent atopia (** **n** ** = 92)**	**p** ** value**
Gender, *n* (%)				
Female	61 (81.3)	27 (71.1)	70 (76.1)	0.45^a^
Male	14 (18.7)	11 (29.8)	22 (23.9)	
Race, *n* (%)				
Caucasian	48 (64.0)	34 (89.5)	65 (70.7)	0.09^a^
African-American	15 (20.0)	2 (5.3)	19 (20.7)	
Asian	8 (10.7)	1 (2.6)	5 (5.4)	
Hispanic	4 (5.3)	0 (0.0)	2 (2.2)	
Unknown	0 (0.0)	1 (2.6)	1 (1.1)	
Age at diagnosis, mean (SD)	31.0 (2.3)	30.1 (3.7)	30.5 (2.0)	0.97^b^
Nail abnormalities, *n* (%)	14 (19.0)	15 (39.5)	9 (9.8)	< 0.001^a^
Hair loss phenotypes, *n* (%)				
Focal/patchy/ophiasis	48 (64.0)	14 (36.8)	42 (45.7)	0.001^c^
Diffuse patchy	16 (21.3)	6 (15.8)	18 (19.6)	
Extensive patchy/universalis	11 (14.7)	18 (47.4)	32 (34.8)	
Patient history of autoimmunity, *n* (%)	25 (33.3)	18 (47.4)	34 (37.0)	0.34^a^
Family history of autoimmunity, *n* (%)	23 (30.7)	12 (31.6)	35 (38.0)	0.57^a^
Family history of AA, *n* (%)	8 (10.7)	1 (2.6)	12 (13.0)	0.20^a^

*Note:* A significance level of 0.017 was used for pairwise ad-hoc comparisons.

Abbreviations: AA, alopecia areata.

^a^Significantly different from AA patients without atopy and eosinophilic phenotype group; *p* values: Pearson's chi-square test.

^b^Significantly different from eosinophilic AA patient group; *p* values: one-way ANOVA.

^c^Significantly different from atopic with AA patients group; *p* values: Kruskal–Wallis test.

**Table 2:** Eosinophilia proportional odds regression results.

**Effect**	**Unadjusted results**		**Adjusted results**	
	**Odds ratio (95% CI)**	**p** ** value**	**Odds ratio (95% CI)**	**p** ** value**
Eosinophilic AA patients vs. AA patients without atopy and eosinophilic phenotype	3.70 (1.73, 7.91)	< 0.001	3.45 (1.59, 747)	0.002
Atopic patients with AA vs. AA patients without atopy and eosinophilic phenotype	2.33 (1.27, 4.25)	0.006	2.31 (1.25, 4.27)	0.008
Eosinophilic AA patients vs. atopic patients with AA	1.59 (0.79, 3.22)	0.20	1.49 (0.72, 3.07)	0.28
Patient history of autoimmunity			2.33 (1.34, 4.05)	0.003
Family history of autoimmunity			1.02 (0.58, 1.80)	0.95

Abbreviations: AA, alopecia areata.

## Data Availability

The corresponding author can provide the data that supports the findings of this study upon request.

## Nomenclature

AA: alopecia areata
AD: atopic dermatitis
AT: alopecia totalis
AtAA: atopic AA patient
AU: alopecia universalis
CnAA: control AA patient
EoAA: eosinophilic AA patient
